# The contributions of vision and haptics to reaching and grasping

**DOI:** 10.3389/fpsyg.2015.01403

**Published:** 2015-09-16

**Authors:** Kayla D. Stone, Claudia L. R. Gonzalez

**Affiliations:** The Brain in Action Laboratory, Department of Kinesiology, University of Lethbridge, LethbridgeAB, Canada

**Keywords:** visually guided, haptics, haptically guided, hand preference, handedness, sensorimotor integration, development, sensory deprivation

## Abstract

This review aims to provide a comprehensive outlook on the sensory (visual and haptic) contributions to reaching and grasping. The focus is on studies in developing children, normal, and neuropsychological populations, and in sensory-deprived individuals. Studies have suggested a right-hand/left-hemisphere specialization for visually guided grasping and a left-hand/right-hemisphere specialization for haptically guided object recognition. This poses the interesting possibility that when vision is not available and grasping relies heavily on the haptic system, there is an advantage to use the left hand. We review the evidence for this possibility and dissect the unique contributions of the visual and haptic systems to grasping. We ultimately discuss how the integration of these two sensory modalities shape hand preference.

## Introduction

Vision is the main sensory system responsible for guiding our actions (Atkinson, 2000), be it searching for one’s keys on a tabletop, navigating through space, recognizing a friend from childhood, or identifying a glass of water to pick it up. But often we forget the pivotal role that haptics plays in completing these actions. Haptics is the perception of combined tactile and kinesthetic inputs during object manipulation and exploration (Grunwald, 2008; Lederman and Klatzky, 2009; Keysers et al., 2010). For instance, upon grasping a glass of water, haptics not only informs where the arm is in space, but also about object properties (e.g., temperature, hardness, weight, texture, and further information about the shape of the cup), which allows for appropriate interaction with the cup (Sober and Sabes, 2003). As can be appreciated from this example, the integration of visual and haptic feedback is central in ensuring efficacy in everyday goal-directed movements. The right- and left-hands, however, do not execute these manual actions to the same extent (Guiard, 1987; Goble and Brown, 2008).

Numerous studies have demonstrated that most individuals have a right-hand preference for visually guided grasping. A significant number of our everyday grasps, however, are haptically guided (e.g., reaching for your keys in a bag). Very little is known about how hand preference for grasping is affected under different sensory conditions. Studies have suggested a right-hand/left-hemisphere specialization for visually guided grasping (Goodale, 1988; Radoeva et al., 2005; Gonzalez et al., 2006; Gonzalez and Goodale, 2009; Janssen et al., 2011; Stone et al., 2013; Flindall and Gonzalez, 2014; Stone and Gonzalez, 2014a,b) and a left-hand/right-hemisphere specialization for haptic processing (Cannon and Benton, 1969; De Renzi et al., 1969; Fontenot and Benton, 1971; Benton et al., 1973; Fagot et al., 1993a,b, 1994; Haaland and Harrington, 1994; Butler et al., 2004; Cote, 2014). This left hand *haptic* advantage might influence hand preference for grasping.

This review will discuss the asymmetrical sensory (visual and haptic) contributions to hand preference for reaching and grasping. First, we will describe these contributions in: (1) developing children, (2) healthy and brain-damaged populations; and (3) sensory-deprived individuals (blind and deafferented). We conclude by proposing that the integration of the two sensory systems modulates hand preference. Very limited attention on this topic, however, has been given to left-handed individuals, so the majority of this review will focus on right-handers. A better understanding of the factors that influence our goal-directed actions will advance our knowledge regarding the organization of the sensorimotor system, provide insight into cerebral asymmetries (including handedness) and serve as the basis for the development of therapeutic devices for the sensory-deprived.

## Sensory Contributions to Hand Preference for Reaching and Grasping: Evidence from Developmental Studies

### Development of Right-Hand Preference for Grasping

It has been suggested that our inclination to use the right hand for manual actions derives from the development of an “asymmetric neuromotor system” in which the left hemisphere develops earlier than the right hemisphere (MacNeilage et al., 2009; Fagard, 2013). Studies in utero have shown that the left hemisphere is larger than the right hemisphere as early as 20 weeks gestation (Hering-Hanit et al., 2001; Fagard, 2013). By 30 weeks gestation, the temporal lobe, the superior sulcus, and the corticospinal tract are larger on the left side than on the right (Dubois et al., 2009; Liu et al., 2010; Kasprian et al., 2011). This asymmetry persists into the first few weeks of life (Gilmore et al., 2007). Furthermore, it has been suggested that postural asymmetries in utero, such as a rightward head-turning preference, also encourage right-hand preference (Michel, 1981; Ververs et al., 1994). It has been speculated that when the head is turned to the right, it is easier for the fetus to bring the right hand to the mouth rather than the left (Fagard, 2013), speculation that could find support from a study showing right-hand preference for sucking as early as 15 weeks gestation (Hepper et al., 1998) which correctly predicted right-handedness into adolescence (Hepper et al., 2005). It is possible that the combination of a more developed left hemisphere and postural preferences in utero may influence right-hand preference for manual actions which become accentuated upon vision taking control of these actions.

Postnatally, studies have documented a right-hand preference for visually guided grasping between 6 and 18 months of age (Carlson and Harris, 1985; Michel et al., 1985, 2006; McCormick and Maurer, 1988; Fagard, 1998; Corbetta and Thelen, 1999; Morange-Majoux et al., 2000; Hinojosa et al., 2003; Fagard and Lockman, 2005; Ronnqvist and Domellof, 2006; Ferre et al., 2010; Jacquet et al., 2012; Nelson et al., 2013; Sacrey et al., 2013; Jacobsohn et al., 2014). Yet, it appears that consistent preference for the right hand is not robust until around 4 years of age (Gesell and Ames, 1947; McManus et al., 1988; Bryden and Roy, 2006; Hill and Khanem, 2009; Sacrey et al., 2013; Gonzalez et al., 2015). Although debate remains regarding *when* this right-hand preference is established, it is clear that the left-hemisphere specialization for visuomotor control develops early in life, and begins to shape hand preference as early as 1 year of age.

Previous studies have recorded hand preference for grasping while infants and children picked up items such as toys (Ramsay, 1980; Fagard and Marks, 2000), building blocks (Sacrey et al., 2013; Gonzalez et al., 2014, 2015), food (Kastner-Koller et al., 2007; Marschik et al., 2008; Sacrey et al., 2013), geometrical shapes (Kotwica et al., 2008), or tools (McManus et al., 1988; Marschik et al., 2008). Noteworthy, in all of these studies infants and children were tested with visual availability, therefore much less is known about hand preference for haptically guided grasping. One study reported that blindfolded-sighted children prefered the use of their right hand for a multitude of actions but the rate of this preference was not reported (Ittyerah, 2000). In the only study (of which we are aware) investigating the effects of vision on hand preference for grasping, 5–8 years old children complete the block-building task (Stone and Gonzalez, 2015b). In the block-building task, particpants are asked to replicate block models from an array of building blocks scattered on a tabletop. Hand use for grasping is documented (Stone et al., 2013). In Stone and Gonzalez (2015b), children were asked to complete the task with and without vision (i.e., while blindfolded). Results showed a marked decrease in right-hand use when vision was unavailable that was comparable to that seen in adults (Stone and Gonzalez, 2015a). This result emphasizes the role that vision plays in right- hand preference for grasping in childhood. It also suggests that haptics can be used to guide reaching and grasping as early as 5 years of age.

### Development of Left-Hand Preference for Haptic Processing

The human hand is sensitive to touch (i.e., cutaneous stimulation) as early as 10.5 weeks gestation. Moreover, by 14 weeks gestation, reflex responses are elicited by stimulation of most of the body surface (Humphrey, 1978). As early as 25 weeks, preterm infants will show cortical evoked responses to cutaneous stimulation and by 26 weeks, they will demonstrate reflexive withdrawal of the foot and leg when stimulated (Andrews and Fitzgerald, 1994). Remarkably, premature infants at 28 weeks demonstrate haptic ability for recognizing novel shapes placed in their left hand (Marcus et al., 2012). By 3 months postnatal, functional magnetic resonance imaging (fMRI) reveals that the cortex and thalamus show a clear, contralateral response to passive cutaneous information presented to each hand (Erberich et al., 2006). Taken together, these reports highlight the early functional development of the somatosensory system.

It has been suggested that there is a division of labor between the hands as early as 4 months of age: the right hand for fine motor movements and the left hand for processing spatial arrangements and haptic information (Morange-Majoux et al., 1997; Morange-Majoux, 2011). Studies on infants and children have shown a left-hand advantage for haptically identifying objects such as wooden cylinders, tactile letters, non-sense or geometrical shapes (Witelson, 1974, 1976; Kalenine et al., 2011; Morange-Majoux, 2011). In one study, 4–6 months old infants were observed manipulating wooden cylinders. The results showed that the left hand spent more time touching and passively exploring the haptic properties of the cylinders than did the right (Morange-Majoux, 2011). It has been suggested that the increased time spent touching the object is due to deeper haptic information processing ability of the left hand (Lhote and Streri, 1998). In fact, infants as young as 2 months of age show the ability to retain haptic information better when that information is exposed to the left hand than when exposed to the right hand (Lhote and Streri, 1998). This result in infants aligns with other studies in early childhood that reported that 2-year-olds display an advantage for visually recognizing novel geometrical shapes that were previously haptically manipulated with the left (but not the right) hand (Rose, 1984). This left-hand advantage for novel object recognition has also been reported in older children (6–12 year olds; Witelson, 1974, 1976). In addition to shape recognition, developmental studies have also demonstrated a robust left-hand advantage for haptically discriminating between different orientations (Brizzolara et al., 1982), as well as for utilizing propriceptive feedback in a trajectory-matching task (Goble et al., 2005). In sum, the pivotal of the right hemisphere for haptics is present and robust early in development.

We have briefly discussed the development of right- and left-hand preferences for visually- and haptically guided movement. The next logical step to gain insight into these sensory asymmetries is to discuss them in healthy and neuropsychological adult populations. The following section reviews the sensory contribtuons to movement in these populations.

## Sensory Contributions to Hand Preference for Reaching and Grasping: Evidence from Healthy and Neuropsychological Populations

Most of the actions we perform on a daily basis are visually guided, such as pointing, pantomiming, gesturing, manipulating, or grasping objects. Multiple studies have concluded that these visuomotor actions are a specialized function of the left-hemisphere (Woodworth, 1899; Fisk and Goodale, 1988; Goodale, 1988; Roy et al., 1994; Heilman et al., 2000; Esparza et al., 2003; Frey et al., 2005a; Johnson-Frey et al., 2005; Radoeva et al., 2005; Serrien et al., 2006; Gonzalez et al., 2007; Janssen et al., 2011; Stone et al., 2013; Sainburg, 2014; Serrien and Sovijarvi-Spape, 2015). In fact, the relationship between visuomotor control (such as during grasping) and the left-hemisphere is so ingrained that simply *viewing* a graspable object (e.g., a piece of fruit, a tool, a toy) elicits a left-hemisphere response in terms of an increase in neural activity (i.e., left premotor cortex; Proverbio et al., 2013) and decreased reaction times when pressing buttons with the right hand (Handy et al., 2003; Netelenbos and Gonzalez, 2015). The following section will review evidence from neuropsychological, kinematic, psychophysical, and natural grasping studies that support the key role of the left hemisphere for *visually* guided actions.

### Left-Hemisphere Specialization for Visually Guided Actions

#### Evidence from Neuropsychological Studies

There is a plethora of brain-damaged patient studies that provide support for a left-hemisphere specialization for visuomotor control (Flowers, 1975; Fisk and Goodale, 1988; Perenin and Vighetto, 1988; Haaland and Harrington, 1994; Haaland et al., 2004; Frey et al., 2005a; Radoeva et al., 2005; Freitas et al., 2011; Mani et al., 2013). Usually, damage to the left hemisphere produces more severe visuomotor impairments than similar damage to the right hemisphere. For example, when individuals were asked to move a cylindrical joystick to a 5 mm target circle those with left-hemisphere damage had slowed peak velocity and longer deceleration when compared to those with right-hemisphere damage (Haaland et al., 2004). Furthermore, studies have shown that damage to the left (but not the right) hemisphere can critically impair goal-directed movements of *both* limbs. For example, individuals with left-hemisphere damage displayed significant impairments in tapping speed with *both* the left and the right hands, but individuals with right-hemisphere damage only showed contralateral (left) hand impairments (Wyke, 1971). Other studies have reported that patients with left-hemisphere damage show significant impairments (including longer execution of the action) for target-directed pointing with the ipsilateral hand when compared to those with right-hemisphere damage who displayed only contralateral deficits (Fisk and Goodale, 1988; Perenin and Vighetto, 1988). Moreover, Haaland and Harrington (1994) tested right- and left-hemisphere stroke patients on a task wherein they were asked to alternately tap between two targets using a stylus as quickly and accurately as possible. While the right-hemisphere group did not differ from controls, the left-hemisphere group was significantly slower than both the control and the right-hemisphere stroke groups. In a more recent study, Mani et al. (2013) asked right- and left-hemisphere stroke patients to move their limbs to different visual targets located on a horizontal plane just above their hand. Only the left-hemisphere stroke patients showed significant impairments in movement trajectory and direction. In sum, the general consensus is that when compared to lesions to the right-hemisphere, left-hemisphere damage leads to more severe impairments in visually guided movement control, in terms of both speed and accuracy, often affecting both limbs.

#### Evidence from Psychophysics and Kinematic Studies

Psychophysical and kinematic studies have confirmed the critical role that vision plays in making appropriate reaching and grasping movements. That is, vision helps to recognize and locate the target, bring the limb to the target, ensure proper reach or grasp configuration, endpoint accuracy, as well as obstacle avoidance (Flowers, 1975; Guiard, 1987; Jakobson and Goodale, 1991; Paulignan et al., 1991; Schiavetto et al., 1993; Jeannerod et al., 1994, 1995; Roy et al., 1994; Jackson et al., 1995; Castiello, 1999; Hopkins et al., 2003; Saunders and Knill, 2003; Westwood et al., 2003; Rand et al., 2007; Keefe and Watt, 2009; Chapman and Goodale, 2010a,b; Babinsky et al., 2012; Tremblay et al., 2013). As early as Woodworth (1899), reported a right-hand advantage for minimizing error during high speed aiming movements, leading him to suggest that the right hand is guided by a ‘superior neural motor center’. Studies involving psychophysical techniques have also reported a left-hemisphere advantage for visuomotor tasks including: finger tapping (Kim et al., 1993); button pressing (Handy et al., 2003; Netelenbos and Gonzalez, 2015; Serrien and Sovijarvi-Spape, 2015); and reaching and pointing (Roy and Elliott, 1986, 1989; Fisk and Goodale, 1988; Goodale, 1988; Elliott et al., 1993, 1995; Roy et al., 1994; van Doorn, 2008). For example, Flowers (1975) compared simple and complex finger tapping actions and found that the right hand was faster and more accurate during the complex finger tapping task. As the complex tapping task requires more precise movements (and thus visual attention), these results suggest that the left hemisphere is better at processing visual feedback during motor movements. Similar results emerge for pointing: Goodale (1988) asked individuals to reach-to-point at different visual targets while the eyes were either (a) fixated at the center of a screen or (b) allowed to freely guide the hand to the target. Individuals were significantly faster at pointing to the target with the right, compared to the left, hand even when the eyes did not guide the hand to the target. Also, this right-hand advantage is not a product of handedness: in a visuomotor illusion task, Gonzalez et al. (2006) showed that in both left- and right-handers, the left hand (and not the right) was affected by the presentation of different visual illusions (for both estimating the length of the object *and* actually grasping it). This effect was later reproduced by Adam et al. (2010), who asked left- and right-handers to reach toward targets on a screen with and without the presence of distracters. Results showed that when the distracters were present, the left hand (regardless of the individual’s handedness) was not only significantly slower at reaching for the target, but often overshot the end point location of the target. Similarly, these results emerge for grasp planning as well. Janssen et al. (2011) had left- and right-handers grasp CD cases in different orientations and found an advantage in planning the movement for right hand (not the left) for both populations.

Kinematic studies have shown a right-hand advantage that is contingent on task demand and/or action type (Roy and Elliott, 1989; Roy et al., 1994; Elliott et al., 1995; van Doorn, 2008; Flindall et al., 2013; Flindall and Gonzalez, 2014). For example, Elliott et al. (1995) asked right-handed individuals to point to small targets, but in some trials, the target suddenly moved to either the left or right side, forcing the individual to correct her/his trajectory. Results showed that participants were better (faster) with their right hand at correcting the movement in response to the target shift. In another study, Flindall et al. (2013) asked right-handed individuals to grasp a glass of water with each hand. Individuals were faster and more accurate at grasping the glass with the right, compared to the left, hand. In further studies, Flindall et al. (2013, 2015) have included left-handed participants to investigate if handedness significantly influences this kinematic advantage (the previous studies only tested right-handed participants). In this series of studies, the right and left hands of right- *and* left-handed individuals were tested in a grasp-to-eat task (Flindall et al., 2015). Participants were asked to grasp for pieces of food to bring to the mouth in order to eat them. The results showed that in both populations, grip aperture was smaller when participants used their right hands. Because smaller grip apertures are typically associated with greater precision, this finding was interpreted as a right-hand advantage for the grasp-to-eat movement regardless of handedness (Flindall et al., 2013, 2015).

Taken together, these studies demonstrate a right-hand advantage for visually guided actions, particularly grasping and suggest that the left hemisphere plays a pivotal role in guiding these actions.

#### Evidence from Neuroimaging Studies

Fewer imaging studies have investigated the role of each hemisphere in visually guided actions due to the challenges of movement artifact associated with executing an action. For instance, in one study using electroencephalography (EEG), Proverbio et al. (2013) asked individuals to view objects that afforded either unimanual or bimanual grasps (e.g., a hammer versus a steering wheel). After only 250 ms of object viewing, the left premotor cortex showed significant activation for both types of grasps, regardless of object orientation (i.e., the hand afforded for the grasp). No manual actions were performed in this study, however. In a fMRI study, Kuhtz-Buschbeck et al. (2001) instructed participants to grasp cubes while inside the scanner. They found that the more precision that was required by the actor to pick up the cube, the stronger the activation of left motor and somatosensory areas. Similar results were reported by Begliomini et al. (2007) and De Sanctis et al. (2013). In these studies, however, only the right hand was tested. When using the left hand, however, results have shown activation in both hemispheres, perhaps due to the increased planning and control associated with using the non-dominant hand (Begliomini et al., 2008, 2015). So it appears that the left-hemisphere is active regardless of which hand is executing an action.

Electroencephalography studies show similar results: participants show increased functional activity of the left (compared to the right) hemisphere for the execution of motor sequences (key presses during a memory-guided task) regardless of hand used or individual handedness (Serrien and Sovijarvi-Spape, 2015). Moreover, fMRI studies have shown preferred left-hemisphere activation for planning an action, observing an action and grasping in both left- and right-handers (Castiello, 2005; Kroliczak and Frey, 2009; Gallivan et al., 2011; Martin et al., 2011). In another study, transcranial magnetic stimulation (TMS) induced motor evoked potentials (MEPs) were recorded while participants watched video clips of left- and right-handed movements (e.g., picking up a thermos to pour into a cup). When the action observed was made with the right hand, participants showed increased MEP activation of both the left *and* the right hands. In contrast, during the observation of left-handed movement, only the left hand showed increased MEP activation (Sartori et al., 2014). Overall, these studies demonstrate a left-hemisphere bias for the visual control of action.

#### Evidence from Natural Grasping Tasks

Studies on hand preference for grasping have shown a right-hand preference for picking up objects such as cards (Bishop et al., 1996; Calvert and Bishop, 1998; Carlier et al., 2006), geometrical 3D shapes (Gabbard et al., 2003), toys (Bryden and Roy, 2006; Sacrey et al., 2013), building blocks (Gonzalez and Goodale, 2009; Stone et al., 2013; Stone and Gonzalez, 2014a,b) and tools (Mamolo et al., 2004, 2006). Furthermore, hand preference tends to remain stable and consistent throughout the lifespan (Gonzalez et al., 2015), save for a slight increase in laterality during adolescence (Bryden and Roy, 2006; Gooderham and Bryden, 2014).

All these studies have controlled for space use in that the objects to be grasped had been equally accessible to either hand. For example Bishop et al. (1996) instructed right-handed individuals to pick up cards arranged in a semi-circle and place them into a box at the midline. A right-hand preference for picking up the cards was observed, even when reaching for the cards in left space. This behavior, although biomechanically costly, is not unusual for right-handed individuals and has been observed in many other studies (Leconte and Fagard, 2004; Bryden and Roy, 2006; Mamolo et al., 2006; Gonzalez et al., 2007; Bryden and Huszczynski, 2011; Stone et al., 2013).

Interestingly, this right-hand preference for grasping does not appear to be linked to handedness. Several studies have shown no hand preference or even a right-hand preference for grasping in left-handers (Gonzalez et al., 2006, 2007; Gonzalez and Goodale, 2009; Gallivan et al., 2011; Stone et al., 2013; Main and Carey, 2014). For instance, Stone et al. (2013) asked right- and left-handed individuals to complete the block-building task (see Gonzalez et al., 2007; Stone et al., 2013). They found that 50% of their left-handed sample showed a preference for grasping with their non-dominant right hand. Similar results have been found by Gonzalez et al. (2007) and Gonzalez and Goodale (2009), who have categorized these left-handers as ‘right-left-handers.’ In other words, some left-handers behave indistinguishably from right-handers in terms of hand selection for grasping.

This prevalent right-hand preference for grasping that includes some (self-identified) left-handers has been attributed to the aforementioned key role of the left-hemisphere in visuomotor control (Kimura, 1977; Fisk and Goodale, 1988; Goodale, 1988; Frey et al., 2005a; Radoeva et al., 2005; Gonzalez et al., 2006, 2007; Serrien et al., 2006; Wang and Sainburg, 2007; Janssen et al., 2011; Stone et al., 2013; Sainburg, 2014). But often we grasp objects in the absence of vision. What do we know about the contributions of haptics to hand preference? In the absence of vision one must rely primarily on the sense of touch (and kinesthesia) to complete a task. It is possible that one might prefer to use the left hand given the known left-hand advantage for haptic processing. The role of the right-hemisphere in haptic processing and haptically guided grasping is discussed next.

### Right-Hemisphere Specialization for Haptic Processing

Even when we are unaware of it, we use haptics for the identification and manipulation of objects (e.g., reaching for keys in a bag, reaching for your cell phone in your pocket, typing on a keyboard). Similar to visually guided movements, when the movement is haptically guided, an individual must find a way to identify and manipulate the object appropriately. Kinematic studies show that when reaching for an object while blindfolded (when compared to while sighted) individuals show larger peak grip apertures (Jakobson and Goodale, 1991; Jackson et al., 1995; Rand et al., 2007; Flindall and Gonzalez, 2014), slower movement times (Schettino et al., 2003; Winges et al., 2003), and a decrease in task accuracy, sometimes knocking over (Wing et al., 1986) or missing the target completely (Babinsky et al., 2012). Furthermore, hand pre-shaping may not occur until tactile contact has been made with the object (Karl et al., 2012; Karl and Whishaw, 2013).

So although movement can still be guided in the absence of vision, the research suggests a decrease in performance under these haptically guided conditions. These studies, however, have only investigated haptically guided grasping with the right hand. It is possible that this decrease in performance during haptically guided movement is not equal between the hands. Perhaps the left hand demonstrates a kinematic advantage compared to the right hand under these conditions. The two studies that have compared the kinematics of the left and right hands for grasping when vision is occluded have shown no advantage for either hand (Grosskopf and Kuhtz-Buschbeck, 2006; Tretriluxana et al., 2008). In these studies, however, vision was either partially occluded, or only occluded after the target had been previously seen. It is possible that total occlusion of vision for the entire experiment would provide different results. So while there is a dearth of kinematic studies that could inform us on manual asymmetries for haptically guided actions, evidence from other sources have shown that in fact, the decrease in performance when vision is occluded is not equal between the hands. This evidence is reviewed below.

#### Evidence from Neuropsychological Studies

Studies in brain-damaged patients provide compelling support for a right-hemisphere specialization for haptic processing (Fontenot and Benton, 1971; Milner and Taylor, 1972; Franco and Sperry, 1977; Kumar, 1977). Kumar (1977) had patients with hemispheric disconnection (i.e., split-brain) complete a tactile version of the Memory for Designs test (see Graham and Kendall, 1960). Using one hand at a time, participants were asked to haptically inspect objects of various shapes and then, using the same hand, to draw whatever shape they had just felt. Participants made significantly fewer errors when using the left hand. The same result is found when patients actively encode geometrical shapes (Franco and Sperry, 1977). Franco and Sperry asked split-brain patients to complete a geometrical shape-matching task. The individuals sat at a table with a curtain in front of them that occluded vision to their hands. Objects were placed in front (within view) and behind the curtain (out of view) and the patient’s job was to haptically match the object behind the curtain with those in front. Results showed that patients were faster and more accurate when they used the left- versus the right-hand. Furthermore, patients with right-hemisphere lesions show bimanual impairments when coding vibrotactile information, whereas left-hemisphere damage leads to only contralesional impairments (Fontenot and Benton, 1971). Taken together, these brain-damaged patient studies reveal a robust left hand/right-hemisphere advantage for haptic processing.

#### Evidence from Studies Involving Psychophysics and Imaging Techniques

Studies involving psychophysics and neuroimaging have also demonstrated a right-hemisphere advantage for haptic processing in both humans (De Renzi et al., 1969; Milner and Taylor, 1972; Benton et al., 1973; Dodds, 1978; Riege et al., 1980; O’Boyle et al., 1987; Wilkinson and Carr, 1987; Fagot et al., 1993a,b, 1994; Butler et al., 2004; Harada et al., 2004; Loayza et al., 2011; Morange-Majoux, 2011; Tomlinson et al., 2011; Cormier and Tremblay, 2013; Stone and Gonzalez, 2014a,b) and non-human primates (Lacreuse and Fragaszy, 1996, 1999). For most of these studies, individuals have been asked to haptically explore, differentiate, or detect geometrical shapes (Franco and Sperry, 1977; Cormier and Tremblay, 2013; Stone and Gonzalez, 2014a,b), non-sense shapes (Dodds, 1978; Fagot et al., 1993a,b, 1994), vibrations (Weinstein, 1978; Rhodes and Schwartz, 1981; Heller et al., 1990; Wiles et al., 1990), or object orientation (Cannon and Benton, 1969; Benton et al., 1973; Varney and Benton, 1975; Brizzolara et al., 1982). For instance, Fagot et al. (1993a,b, 1994) had individuals haptically explore different cubes either unimanually or bimanually and measured accuracy during a recognition test. When both hands were used, individuals were more accurate at identifying the cubes explored more with the left, rather than the right hand (Fagot et al., 1993b; Lacreuse et al., 1996). When one hand was used, it was found that individuals used the left hand to cover more surface area per cube and touched more cubes overall during this haptic recognition task (Fagot et al., 1993a, 1994). Furthermore, even when the experimenter moves an object across the palms of the participant (rather than the participant actively exploring it), the left hand is more accurate at detecting differences between stimuli (Benton et al., 1973). Aligned with these findings, in a more recent study, individuals were asked to assess the curvature of different virtual contours (Squeri et al., 2012). While grasping the handles of a manipulandum, the hands were passively moved along a curved pathway “as if exploring the smooth surface of a round object.” Results showed that the left hand was more sensitive to detecting differences in curvature. The authors conclude that the left hand produces more precise haptic estimates than does the right hand. Finally, it appears that the right-hemisphere’s role in haptic processing is not dependent on individual handedness. A study found that the left thumb is more accurate than the right thumb (for both left- and right-handers) in terms of detecting sense position, which requires the processing of haptic feedback (Riolo-Quinn, 1991).

Imaging studies have shown support for the theory of a right-hemisphere specialization for haptics. Using fMRI, Harada et al. (2004) found that regardless of hand, when an individual’s fingers were passively moved across Braille letters there was increased activation in the right hemisphere (frontal and parietal areas) when compared to the left hemisphere. Loayza et al. (2011) applied vibrations to the left and right hands of participants while undergoing an fMRI scan. Results revealed increased right-hemisphere activation (fronto-parietal areas) for detecting stimulus location on the hand, regardless of the hand that was stimulated. Furthermore, Cormier and Tremblay (2013) showed that right-handers exhibit increased corticomotor excitability in the right-hemisphere when haptically judging the thickness of a metal plate with the left hand (compared to the left-hemisphere/right-hand). Together, these studies illustrate the unique role of the right hemisphere in haptic processing.

Finally, it is possible that this left-hand/right-hemisphere advantage for haptic processing is a co-product of the right hemisphere’s specialization for global processing (Heller and Clyburn, 1993; Lux et al., 2004; Tomlinson et al., 2011; Langerak et al., 2013). For instance, Langerak et al. (2013) showed that participants are faster at responding to objects near the left hand (compared to the right hand) when discriminating between targets at the global (versus local) level. Future studies could investigate this possibility in the absence of vision.

#### Evidence from Natural Grasping Studies

**FIGURE 1:**
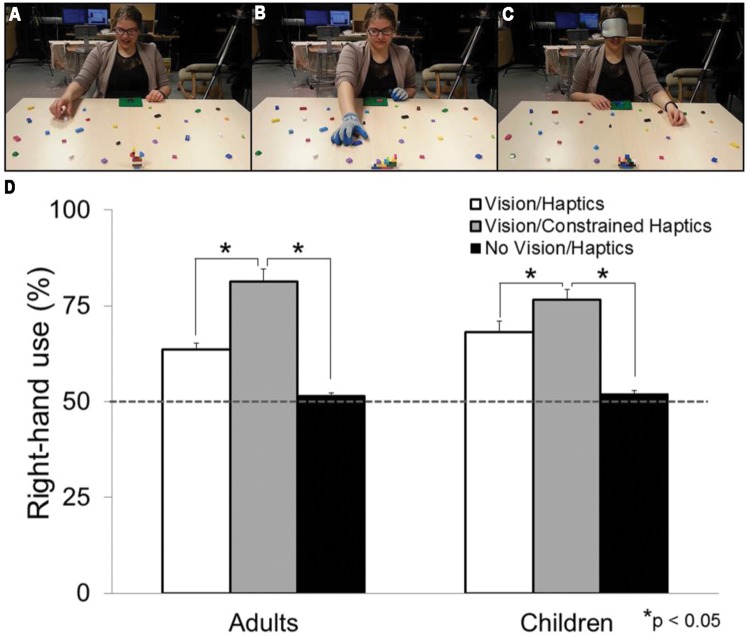
**Experimental set-up and results from Stone and Gonzalez (2014a,b, 2015a,b).** Photographs of participants completing **(A)** the Vision/Haptics condition **(B)** the Vision/Constrained-Haptics condition (note that the participant is wearing a pair of gloves) and **(C)** the No Vision/Haptics condition (note that the participant is wearing a blindfold) **(D)** Graph demonstrating right-hand use for grasping in percentage for the three sensory conditions in children and adults. White bars represent the Vision/Haptics condition. Gray bars represent the Vision/Constrained-Haptics condition. Black bars represent the No Vision/Haptics condition. The gray dashed line denotes 50% right-hand use (or equal use of each hand). Note the significant difference within sensory conditions.

Because vision is unavailable during haptically guided tasks, individuals will use exploratory procedures (EP) to extract relevant information about the object(s) or stimuli. EPs are stereotyped patterns of hand movements used to extract object properties and features during haptic object recognition (Lederman and Klatzky, 1987, 2009). There are six observable types of EPs, each specialized for encoding specific haptic properties. These include: lateral motion (for texture); unsupported holding (for weight); pressure (for hardness); enclosure (for global shape and volume); contour following (for global shape and exact shape); and static contact (for temperature). It has been concluded that the most effective way to haptically process an object is to grasp it which at minimum combines enclosure, static contact, and unsupported holding (Lederman and Klatzky, 1990, 2009). If grasping is the most effective method to use for haptic object recognition, then grasping could be used as a model to investigate hemispheric asymmetries in haptic processing. Yet, this is rarely the case. In a series of studies, Stone and Gonzalez (2014a,b) asked right-handed individuals to grasp building blocks in order to replicate different 3D models (i.e., the block-building task) while sighted (see **Figure 1A**) and while blindfolded (see **Figure 1C**). The hand selected for picking up each block was assessed. Although a right-hand preference was observed during the visually guided portion of the task, there was a significant increase in left-hand use when the task was haptically guided (i.e., while blindfolded; see **Figure 1D**). Because without vision, individuals must use haptics to guide their actions (and in turn manipulate and discriminate between the different types of building blocks), the authors attributed their finding to a left-hand/right-hemisphere specialization for haptic processing. What is more, if participants haptically manipulated the building blocks in a container 5 min prior to the block-building task, they showed an even greater preference for the left hand when completing the task. It appears that 5 min of an added ‘haptic experience’ increases the preference to use the left hand. If this is the case, how would a lifetime of haptic experience affect hand preference for grasping? To address this question, investigations involving congenitally blind (CB) individuals are discussed in the following section.

## Sensory Contributions to Hand Preference for Reaching and Grasping: Evidence from Sensory-Deprived Populations

### Congenitally Blind Individuals

One population that inarguably has a lifetime of haptic experience is CB individuals. CB are those who were born without sight, or lost sight shortly thereafter, and therefore have no recollection of having a visual experience (Thinus-Blanc and Gaunet, 1997). Most cases of congenital blindness are due to dysfunctional development of the retina and/or optic nerve (Mccolm and Fleck, 2001). In turn, the CB rely on their other senses (mainly haptics and audition) to guide their movements. Many studies have compared haptics in CB versus sighted individuals (Hermelin and O’Connor, 1971; Millar, 1974; Heller et al., 1996; Ittyerah, 2000, 2009; Ittyerah and Marks, 2007; Collignon and De Volder, 2009; Theurel et al., 2012, 2013). For example, Theurel et al. (2012) asked CB and blindfolded-sighted individuals to haptically discriminate between different geometrical shapes including common (square, triangle, rectangle) and non-sense shapes. The CB were more efficient in their EPs for identifying the shapes, and could identify non-sense shapes just as easily as the common shapes. Blindfolded-sighted individuals, on the other hand, were only proficient at identifying common shapes. These results align with Postma et al. (2007) who found that, in comparison to blindfolded controls, blind individuals were more accurate in a cut-out shape identification and matching task. Neither of these studies, however, assessed hand differences for haptics. For the most part, participants were asked to use their dominant hand or both hands to complete the task. This scenario does not provide any information about differences in haptic ability between the two hands in blind individuals.

Hand preference in CB individuals has been seldom investigated. The few studies that have assessed hand preference in the CB have been subjective (i.e., through the use of questionnaires or interviews) and/or have focused mainly on children. Ittyerah (2000, 2009), for example, had CB children between the ages of 6 and 15 years of age complete a multitude of tasks such as putting beads in a jar, cutting paper with scissors, picking up a pen, or throwing a ball. Both CB and blindfolded-sighted children displayed a preference (of similar extent) to use the right hand. The children were also asked to sort items such as cards, buttons, tokens, and paper clips, and it was revealed that CB children were significantly faster at sorting the objects with the left hand. These results highlight that although there may be a preference for the right hand for certain tasks, the left hand still plays a critical role in haptically identifying objects (i.e., in this case, to sort). Yet, another study by Caliskan and Dane (2009) showed that CB children between the ages of 7 and 12 years were more likely to be left-handed than were sighted children, though these results were based on a questionnaire. Using questionnaires, studies have also reported CB adults to be right-handed (Nava et al., 2013; Argyropoulos et al., 2014). None of the adult studies specifically assessed hand preference for *grasping*. Closing this gap, Stone and Gonzalez (2014b) asked CB, sighted, and blindfolded-sighted individuals to complete the block-building task and recorded hand selection for grasping the blocks. As in Stone and Gonzalez (2014a), the blindfolded-sighted group used their left hands significantly more than the sighted group highlighting the difference in hand use between visually guided and haptically guided grasping. Interestingly, the CB group showed a right-hand preference for grasping that was indistinguishable from that of the sighted participants. So even though the CB had a lifetime of haptic experience, they did not demonstrate a left-hand preference for grasping. Instead, their behavior was similar to that of sighted individuals. The question remains: why is this the case if sighted and CB rely on different sensory modalities to complete the task?.

One possibility might be related to similar processing in the ventral and dorsal visual streams found in sighted and CB individuals. These pathways project from primary visual cortex to the inferior temporal lobe (the ventral stream) and to the posterior parietal cortex (PPC; the dorsal stream; Goodale and Milner, 1992). The ventral (vision-for-perception) stream is responsible for object identification, or knowing ‘what’ an object is, whereas the dorsal (vision-for-action) stream is responsible for the visuomotor transformation and control of actions, or knowing ‘where’ the object is in space and ‘how’ to interact with it (e.g., manipulate and grasp). Although CB individuals have a lifetime without visual input, surprisingly, their dorsal, and ventral “visual” streams are preserved (Pietrini et al., 2004; Poirier et al., 2006; Amedi et al., 2007; Fiehler et al., 2009; Mahon et al., 2009; Ricciardi et al., 2009; Renier et al., 2010; Collignon et al., 2011; Reich et al., 2011; Striem-Amit et al., 2012). The lateral occipital complex (LOC) is an area located in the ventral stream that is responsible for both the visual and haptic identification of object shape (Amedi et al., 2001; James et al., 2002, 2007; Pietrini et al., 2004; Zhang et al., 2004; Stilla and Sathian, 2008). In an fMRI study, sighted and CB individuals were asked to identify different common objects (e.g., shoe, water bottle) using both hands. Both groups showed robust activation of area LOC during haptic recognition, even though the CB had never visually experienced the object before (Pietrini et al., 2004). Moreover, hearing auditory properties (e.g., ‘crinkling’ and ‘crumbling’) of material objects also elicit ventral stream activation in the CB (Arnott et al., 2008). These results demonstrate that object recognition in the ventral visual stream remains functionally specialized even without visual experience. Similarly, the dorsal visual stream is also functionally specialized in CB individuals. In an fMRI study, CB and blindfolded-sighted participants were asked to trace different line patterns using a stylus in their right hand to investigate brain activation during movement of the limbs and hands (Fiehler et al., 2008). The same dorsal stream areas were activated in both groups during this task, primarily the anterior intraparietal sulcus (aIPS) and superior parietal lobe. Moreover, the human middle temporal area (area hMT+), a portion of the dorsal stream that is responsible for processing motion, shows overlapping activation in sighted and blind individuals, be it for visual or tactile motion (Ricciardi et al., 2007).

Because similar visual areas are activated in both CB and sighted individuals for perception and action, it makes sense that the CB group behaved similarly to the sighted group in terms of hand selection during the grasping task (Stone and Gonzalez, 2014b). Further supporting this notion, kinematic studies have shown that like sighted individuals, CB showed: size-appropriate grip scaling when grasping different sized objects (Castiello et al., 1993); similar hand orientation in a posting task (Gosselin-Kessiby et al., 2009); and similar grip apertures for grasping (Karl et al., 2012). In sum, early loss of vision in humans appears to result in reorganization that affords similar grasping profiles as those observed among normally sighted individuals.

### Deafferented Individuals

Another population that could provide insight into the contributions of sensory information to grasping is individuals with deafferentation. Deafferentation is a rare condition that occurs from the degeneration or loss of the large afferent nerve cells that convey information about touch and/or position sense (Cole and Sedgwick, 1992; Proske and Gandevia, 2012). Motor control in the deafferented has been previously investigated (Rothwell et al., 1982; Cooke et al., 1985; Forget and Lamarre, 1987; Cole and Sedgwick, 1992; Gentilucci et al., 1994; Nowak et al., 2004; Travieso and Lederman, 2007; Hermsdorfer et al., 2008; Sens et al., 2013). A seminal investigation on deafferentation and motor control was conducted in 1982 with patient GO, who had severe peripheral sensory neuropathy induced by influenza (Rothwell et al., 1982). Patient GO had an extreme reduction in vibration and temperature detection, reduced response to skin pricks, and severe impairments in light touch recognition. Although GO’s hands were “relatively useless to him in daily life” (Rothwell et al., 1982, p. 515) he was still able to complete a multitude of manual actions. For instance, he was able to accurately produce different levels of force on his thumb pad when asked. He was also able to accurately complete simple finger movements (e.g., outline shapes, tap his fingers, or wave his hands). These manual actions, however, were guided entirely by vision. After approximately 30 s without visual *or* haptic input, he could no longer complete these types of tasks. Because GO was a right-handed man, most of the testing focused on the use of his right hand, thus not allowing for an analysis of possible asymmetries in the contribution of haptics to manual actions. With respect to the few studies that have investigated reach-to-grasp movements, it has been reported that deafferented individuals show overall longer movement times and immense variability in their movements (Gentilucci et al., 1994; Jackson et al., 2000, 2002). Jackson et al. (2002) investigated grasping in a deafferented individual (patient DB) with no sense of touch in her left arm (yet her proprioception was intact). Patient DB was asked to reach for wooden dowels using one hand or both hands. Immediately after the reach was initiated, vision was occluded via liquid crystal goggles. Results revealed that during unimanual trials, DB’s left and right hands took the same amount of time to reach the target. However, during the bimanual trials, the left hand (when compared to the right hand) took significantly longer and was considerably more variable in reaching for its left-side target. This is in contrast to controls who showed no difference in movement time between the limbs for bimanual actions. When the task was visually guided, her movement was virtually unimpaired (Jackson et al., 2000). Yet only DB’s left arm affected by her deafferented condition; perhaps if both arms were affected manual asymmetries might emerge for motor actions. Gentilucci et al. (1994) assessed reach-to-grasp movements in a bilaterally deafferented individual (who had no sense of touch *or* proprioception from her shoulders down). She took significantly longer than controls to close her fingers over the target (a sphere), while also displaying immense variability in these movements. Only the right hand was tested, however, not allowing for a comparison between the hands. Nonetheless, these studies highlight the importance of haptic feedback during reach-to-grasp movements.

One method of inducing deafferentation in healthy individuals is via Temporary Functional Deafferentation (TFD), which creates a pharmacological blockade of peripheral nerve transmission (Sens et al., 2012; Opsommer et al., 2013). For the most part, this method has been used in stroke rehabilitation (Werhahn et al., 2002; Weiss et al., 2011; Sens et al., 2012, 2013; Opsommer et al., 2013), however, some studies have documented the effects of TFD in healthy individuals. In stroke rehabilitation, using an anesthetic cream on the affected arm enhances performance on a variety of tactile and motor tasks (Sens et al., 2013). In healthy individuals, a few studies have shown enhanced sensorimotor performance with anesthetic-cream based TFD (Bjorkman et al., 2004, 2009; Petoe et al., 2013) whereas one study showed no effect of TFD on sensorimotor performance (Sens et al., 2013). It should be noted that in these studies, TFD cream was applied to the forearm and not to the hands. As argued by Bjorkman et al. (2004) the enhanced sensorimotor function may be due to an expansion of cortical sensory representation of the hand which is adjacent to the forearm. With respect to the hands, however, it has been shown that *tourniquet*-induced anethseia of the right hand improves sensorimotor function (i.e., grip stength., tactile discimination, tactile acuity) of the left hand (Werhahn et al., 2002; Bjorkman et al., 2004). This last result demonstrates that hand function can be enhanced by temporarily inducing deafferenation in the contralateral arm (as the previous studies tested the ipilateral arm). No study to our knowledge has used this method of transient deafferentation applied to both of the hands to investigate hand preference and/or performance for grasping or other sensorimotor tasks.

Although motor control has been assessed in the deafferented, there is a dearth of information on *hand preference* for grasping in this population. We speculate, however, that bilaterally deafferented patients would favor the right hand for grasping. If haptics is a specialization of the left hand, then in the absence of that sense, one would resort to using the right hand because as argued in the previous section, when grasping with vision the right hand is preferred for grasping. Consistent with this speculation, a recent study found that constraining haptics (i.e., by asking participants to wear a pair of textured, fitted gloves; see **Figure 1B**) during a grasping task, results in a decrease of left-hand use to the point that the right hand is used almost exclusively (Stone and Gonzalez, 2015a; see **Figure 1D**).

Together, studies in the sensory deprived (i.e., CB and deafferented) provide a glimpse into the asymmetric contributions of the visual and haptic systems to sensorimotor control, and by no means are the results conclusive. There is ample opportunity to further this knowledge using these populations, and by including related populations, such as late blind individuals or patients with tactile agnosia, tactile apraxia, or autotopagnosia.

## Integration of Hemispheric Specializations

Overall, the literature suggests a left-hemisphere specialization for visually guided movements and a right-hemisphere specialization for haptic processing. These functional asymmetries tend to be developed in childhood, and possibly even infancy. On a moment-to-moment basis, vision and haptics work together to create a perception of the world and the ability to act upon it. It has been argued that concurrent use of visual and haptic information provides the best means to recognize an object (Woods and Newell, 2004). Many studies have investigated the relationship between these two sensory systems and have demonstrated their interconnectedness at both the behavioral and neural levels (Reales and Ballesteros, 1999; Sadato et al., 2002; Norman et al., 2004, 2008; Woods and Newell, 2004; Millar and Al-Attar, 2005; Held, 2009; Volcic et al., 2010; Hupp and Sloutsky, 2011; Lacey and Sathian, 2011, 2014; Xu et al., 2012; Lawson et al., 2013; Rognini et al., 2013; Wallraven et al., 2014; Wesslein et al., 2014).

### Evidence from Behavioral Studies

One way to understand the imbricated relationship between vision and haptics to manual actions is to investigate facilitation or interference effects when the senses are combined or isolated. Millar and Al-Attar (2005) explored the extent to which vision facilitates haptic processing. Using the right index finger, participants were asked remember spatial landmarks on a tactile map while visual availability was manipulated (i.e., no vision, tunnel vision, peripheral vision, or full vision). Results showed that having vision increased performance on the task, even if it was just tunnel or peripheral vision. Moreover, Tipper et al. (1998) found that having vision of the hand (via a computer screen) during a tactile recognition (i.e., vibration) task significantly improved participant’s response time. This finding has been replicated in other studies (Sambo et al., 2012; Wesslein et al., 2014). With respect to visuo-haptic interference, Eimer and Driver (2000) found that spatial attention to a tactile cue (i.e., blunt metal rod tapped on fingertip) interferes with subsequent attention to a visual cue (i.e., green lit circle), but not vice-versa. This result suggests a unidirectional relationship between vision and touch, at least in terms of spatial attention. However, in a study that investigated the bidirectional contributions of vision and haptics to grasping, participants were asked to reach-to-grasp various sized wooden blocks using the right hand (Pettypiece et al., 2010). Concurrently, participants gripped a wooden block with the left hand that was either the same (congruent) or a different (incongruent) size as the block they were instructed to grasp with the right hand. When the object in the left hand was incongruent (larger), participants opened their right hand significantly wider prior to grasp onset. That is, even though the participant could *see* the object they were grasping, haptic information in the left hand interfered with the kinematics of the right. Therefore, although vision enhances performance on a haptic task, haptic information can also affect performance on a visual task.

One theory supporting this integrated relationship is known as the ‘optimal integration theory’ or sometimes referred to as ‘the sensory weighting hypothesis’ (Ernst and Banks, 2002; Spence and Walton, 2005; Helbig and Ernst, 2007). This theory posits that during a task that involves sensory competition (such as the presence of both vision and touch), humans will rely on whichever domain provides optimal information to complete the task. For example, if you are looking for your cup of coffee in a well-lit room, vision will arguably provide more relevant information than haptics. In contrast, if the room is poorly lit, haptics might assume a more dominant role in identifying the cup. In one study, Ernst and Banks (2002) presented individuals with two bars and asked them to indicate which bar was taller. Participants explored the bars either visually and haptically. The visual scene was manipulated in order to investigate when individuals would switch to relying on one sense versus the other. They found that when the visual scene was “noisy” (i.e., with distractors), performance tended to rely more on the haptic domain, demonstrating that humans integrate sensory information in a statistically optimal fashion (Ernst and Banks, 2002). Further support for optimal integration of visual and haptic cues is evident in a recent report by Kandula et al. (2015). They reported that individuals were faster to respond to tactile stimuli when it was congruent (rather than incongruent) with incoming visual stimuli. Participants watched a video of a hand coming toward their face while vibrotactile stimulation was applied to the cheek. When the stimulation matched that of the projected hand path coming toward the cheek, participants were significantly faster at responding to the vibration. Similar results with respect to congruent/incongruent visual and haptic stimulation were reported by Gray and Tan (2002). These behavioral studies highlight an integrated, inter-sensory relationship between the visual and haptic systems.

### Evidence from Neuroimaging Studies

This integrated relationship has also been shown at the neural level in areas including the occipital and parietal cortices. For instance, TMS to the occipital cortex of healthy individuals not only impairs visual perception (e.g., Beckers and Homberg, 1991) but also tactile processing (Zangaladze et al., 1999). In fact, patient DF, an individual with ventral visual stream damage (mainly in the occipital areas) causing visual-form agnosia, shows extreme impairments in visual as well as in haptic object recognition (James et al., 2006). fMRI studies in healthy individuals have shown activation of the middle and lateral occipital areas during visual and haptic recognition of 3D non-sense objects (James et al., 2002). The LOC has been coined a ‘visuo-haptic shape identification’ area (Amedi et al., 2001; Grill-Spector et al., 2001; Lacey et al., 2009). For instance, in an fMRI study, Amedi et al. (2001) asked individuals to visually and haptically identify different 3D common objects (e.g., fork, syringe). Results showed significant object-related activation in area LOC for both visual and haptic identification (Amedi et al., 2001). Furthermore, the right LOC shows greater activation when haptic processing is completed with the left hand, compared to activation of the left LOC when using the right hand (Yalachkov et al., 2015). Taken together, these studies highlight the role of occipital areas in visuo-tactile processing.

The parietal cortex also plays a key role in multisensory processing. In fact, simply observing someone else being touched has been shown to activate areas such as the secondary somatosensory cortex (SII; Keysers et al., 2004) and the PPC (Chan and Baker, 2015). The PPC is implicated in both the visual (Goodale and Milner, 1992) and haptic (Dijkerman and de Haan, 2007) dorsal streams, which are responsible for how we interact with objects. The visual dorsal stream, which projects from primary visual cortex to PPC, aids in the visuomotor transformation and control of actions. The haptic dorsal stream which projects from SI and SII also to the PPC assists in the haptic-motor transformation of information for action. Both the dorsal-visual and the dorsal-haptic streams are responsible for transforming information about an object’s features (e.g., size, orientation, location) for appropriate grasp and manipulation. With respect to integration of vision and haptics, fMRI studies have shown activation in the aIPS, an area implicated in grasping (Binkofski et al., 1998; Grezes et al., 2003; Castiello, 2005; Frey et al., 2005b, 2015; Culham and Valyear, 2006; Gallivan and Culham, 2015) during both visual and tactile object recognition (Grefkes et al., 2002; Tal and Amedi, 2009). Overall, it is clear that parietal areas play a role in the integration of vision and touch.

Not surprisingly, these same occipito-parietal areas responsible for visual and haptic integration have also been implicated in grasping. Studies investigating this sensory integration for grasping are discussed below. We conclude by proposing a model of how vision and haptics shape hand preference for grasping.

### Sensory Integration for Grasping

The combined role of *both* vision and haptics during reach-to-grasp movements has been investigated in few studies (Kritikos and Beresford, 2002; Chang et al., 2008; Pettypiece et al., 2010; Endo et al., 2011; Buckingham et al., 2012). In one study, using the size-weight illusion (an illusion in which objects of similar size differ in weight, and objects of identical weight differ in size) participants were asked to lift with one hand objects that were resting on the palm of the other hand or on a tabletop. Results showed more accurate lifting forces when the object rested on the hand (objects only rested in the left hand) presumably because the left hand provided helpful haptic feedback to guide the lift made by the right hand (Chang et al., 2008). This study unfortunately only tested the right hand for lifting (left hand for resting). In a different study that tested both hands, Kritikos and Beresford (2002) investigated the effects of visual and haptic information on grasp formation. Individuals were asked to make a visually guided grasp toward a target object (rubber ball) with one hand while the other hand held an unseen distractor object (that was either smaller, larger, or the same size as the target object). Because the distractor was not seen but instead only felt, the authors were able to investigate if irrelevant haptic information in one hand affected the grasping parameters of the other. Intriguingly, grasp kinematics were only affected when the left hand held the distractor object. The authors concluded that irrelevant haptic information has an influence on visuomotor control and argued that the left-handed kinematics were not affected by holding an object in the right hand because the left-handed kinematics were already quite variable. Alternatively, one could speculate that because the left hand is better at haptic processing, holding an object in the left hand would have a greater influence on the actions of the right hand than would the opposite. If this hypothesis were correct, the results would suggest that the left-hemisphere specialization for visually guided grasping can be easily influenced by the right-hemisphere’s role in haptics. This possibility, and whether haptically guided grasping could be influenced by visual information, warrants further investigation.

### A Model of How Visuo-Haptic Integration Influences Hand Preference for Grasping

In the only study (to our knowledge) that has investigated how vision *and* haptics modulate hand preference for grasping, right- and left-handed adults were asked to complete the block-building task under four conditions: Vision/Haptics, No Vision/Haptics, Vision/Constrained Haptics, No Vision/Constrained Haptics (Stone and Gonzalez, 2015a). In the No Vision conditions participants wore a blindfold and in the Constrained Haptics they wore a pair of textured, fitted gloves (see **Figure 1B**). Results showed a right-hand preference (∼65%) for grasping when vision and haptics were both available (Vision/Haptics), replicating numerous other studies (Bryden et al., 2000; Cavill and Bryden, 2003; Gonzalez et al., 2007, 2015; Gonzalez and Goodale, 2009; Stone et al., 2013; Main and Carey, 2014; Stone and Gonzalez, 2014a,b). When vision was occluded, and the task was haptically guided (No Vision/Haptics condition), there was a significant increase in *left*-hand use, to the point where the right and left hands were used to the same extent (∼50% left-hand use). This result also replicated the findings of Stone and Gonzalez (2014a,b). Interestingly, when vision was available but haptics was constrained (Vision/Constrained Haptics), the right hand was used almost exclusively (∼80%, see **Figure 1D**). These results strongly suggest the interconnectedness of the visual and haptic systems in shaping hand preference for grasping in which both sensory systems, albeit in opposite directions, contribute to this preference. What is more, similar results were also found in children as early as 5 years of age (Stone and Gonzalez, 2015b) suggesting that vision and haptics have a modulatory effect on hand preference since early development.

If vision and haptics both contribute to shaping hand preference for grasping (from opposite hemispheres), why do individuals still present with a right-hand preference (∼65%)? One possibility is that handedness plays a role. However, numerous studies have shown that left-handers are not the mirror image of right-handers and in fact, as a population left-handers do not show a hand preference for grasping in some studies (Mamolo et al., 2005; Bryden et al., 2011; Stone et al., 2013; Main and Carey, 2014; Stone and Gonzalez, 2015a). Moreover, hand preference remained unchanged for left-handers in response to the visual and haptic manipulations in Stone and Gonzalez (2015b). This finding is supported by Tomlinson et al. (2011) who found that left-handers show much weaker lateralization during a haptic task than right-handers, as well as by Pool et al. (2015) who found that left-handers have significantly lower interhemispheric functional connectivity between sensorimotor areas. Another possibility is that higher rates of right-hand use in the presence of both vision and haptics, are a reflection of the type of grasping actions that we execute. Studies have shown kinematic differences in seemingly similar actions that only differ in the ultimate goal of the action [e.g., grasp-to-place vs. grasp-to-throw (Armbruster and Spijkers, 2006); grasp-to-place versus grasp-to-eat (Flindall and Gonzalez, 2014; Flindall et al., 2015)]. It remains to be shown if haptically guided grasping movements that require only identification of an object (i.e., grasp-to-identify) generate higher rates of left-hand use. In our studies using the block-building task, participants are not only required to haptically identify the blocks that constitute the sample model, but are also required to manipulate and assemble the pieces to successfully construct a replica (grasp-to-construct). It is possible that hand preference would be different for these two very different types of grasps (i.e., grasp-to-identify versus grasp-to-construct). Observations from our lab may lend support to this idea. Although not specifically investigated, in Stone and Gonzalez (2015a) participants made more grasp-to-identify movements with the left hand than they did with the right hand. This suggests that hand preference is sensitive to the intent behind a grasp. Experiments specifically testing this suggestion are underway. Furthermore, studies investigating the role of handedness in the interplay of visual and haptic information are necessary to gain a comprehensive view on cerebral asymmetries for sensorimotor processing.

Finally, a viable model to explain hand preference for grasping would be to frame it around an evolutionary scenario. Studies have shown that non-human primates exhibit a left-hand preference for haptic discrimination (Fagot et al., 1991; Lacreuse and Fragaszy, 1996, 1997, 1999; Laska, 1996; Parr et al., 1997; Spinozzi and Cacchiarelli, 2000) and a right-hand preference for visually guided reaching and grasping (MacNeilage et al., 1987; Hopkins et al., 1993; Diamond and McGrew, 1994; Hopkins, 1995). For example, Lacreuse and Fragaszy (1999) observed Capuchins searching for sunflower seeds in the crevices of 12 clay objects using haptics, similar to a ‘grasp-to-identify’ action. Capuchins showed a robust preference to use the left hand during this task. The authors suggested that “the left-hand preference for the haptic task may reflect a hemispheric specialization to integrate the spatial and motor components of an action” (Lacreuse and Fragaszy, 1999; p. 65). Conversely, during a visually guided reach-to-eat task, Spinozzi et al. (1998) showed that Capuchins show a preference to grasp with the right hand, an preference found in chimpanzees as well (Hopkins and Fernández-Carriba, 2000; Hopkins et al., 2013). So it is plausible that these functional asymmetries were present in our common ancestors and thus passed through our lineage. Since the majority of grasps in primates are not specifically to identify an object (vision usually enables object identification) then recruitment of the left hand for grasping occurs less frequently.

### Conclusion

The literature suggests a left-hemisphere specialization for visuomotor control, particularly for visually guided grasping and a right-hemisphere specialization for haptic processing. We speculate that these sensory-modality specific asymmetries integrate to contribute to hand preference during grasping (even early in development). During visually guided grasping, a dominant role for the right-hand/left-hemisphere presents, but during haptically guided grasping, a trend to rely more on the left-hand/right-hemisphere emerges. Taken together, the interplay of these two systems allows for effective sensorimotor control.

## Conflict of Interest Statement

The authors declare that the research was conducted in the absence of any commercial or financial relationships that could be construed as a potential conflict of interest.
